# Childhood Gender Diversity and Mental Health: Protocol for the Longitudinal, Observational Gender Journey Project

**DOI:** 10.2196/55558

**Published:** 2024-08-09

**Authors:** Marco A Hidalgo, Diane Chen, Amy C Tishelman, Johanna Olson-Kennedy, Yee-Ming Chan, Robert Garofalo, Hanno Petras, Stephen M Rosenthal, Diane Ehrensaft

**Affiliations:** 1 Internal Medicine-Pediatrics and Preventive Medicine Section Division of General Internal Medicine and Health Services Research, Department of Medicine David Geffen School of Medicine at University of California Los Angeles Los Angeles, CA United States; 2 Gender Health Program UCLA Health University of California Los Angeles Los Angeles, CA United States; 3 Gender & Sex Development Program Potocsnak Family Division of Adolescent and Young Adult Medicine Ann & Robert H. Lurie Children’s Hospital of Chicago Chicago, IL United States; 4 Department of Pediatrics Feinberg School of Medicine Northwestern University Chicago, IL United States; 5 Pritzker Department of Psychiatry and Behavioral Health Ann & Robert H. Lurie Children's Hospital of Chicago Chicago, IL United States; 6 Department of Psychiatry and Behavioral Sciences Feinberg School of Medicine Northwestern University Chicago, IL United States; 7 Department of Psychology and Neuroscience Boston College Chestnut Hill, MA United States; 8 Center for Transyouth Health and Development Division of Adolescent and Young Adult Medicine Children's Hospital Los Angeles Los Angeles, CA United States; 9 Department of Pediatrics Keck School of Medicine University of Southern California Los Angeles, CA United States; 10 Division of Endocrinology Department of Pediatrics Boston Children's Hospital Boston, MA United States; 11 Department of Pediatrics Harvard Medical School Boston, MA United States; 12 National Capital Region Center Pacific Institute for Research and Evaluation Beltsville, MD United States; 13 Child and Adolescent Gender Center Benioff Children’s Hospital University of California San Francisco San Francisco, CA United States; 14 Department of Pediatrics Division of Pediatric Endocrinology University of California San Francisco San Francisco, CA United States

**Keywords:** transgender, prepubertal, mental health, gender identity, development, childhood, protocol, observational, gender, children, gender-diverse, United States, dysphoria, TGD, transgender, nonbinary, and gender-diverse, symptoms, diagnoses

## Abstract

**Background:**

Prepubertal transgender, nonbinary, and gender-diverse (TGD) children (ie, those asserting gender identity, expressing gender-role behavior outside of culturally defined norms for their sex registered at birth, or both) are presenting in greater numbers to pediatric gender clinics across the United States and abroad. A large subset of TGD children experiences gender dysphoria, that is, distress that arises from the incongruence between gender identity and sex registered at birth. A lack of consensus exists regarding care for prepubertal TGD children due, in part, to a dearth of empirical research on longitudinal developmental trajectories of gender identity, role behavior, and gender dysphoria (when present).

**Objective:**

The objective of this National Institutes of Health–funded study is to provide evidence to inform clinical care for prepubertal TGD children by establishing a US longitudinal cohort (N=248) of prepubertal TGD children and their caregivers that is followed prospectively at 6-month intervals across 18 months.

**Methods:**

At each timepoint, clinical and behavioral data are collected via web-based visit from child and caregiver reporters. Latent class analysis, among other methods, is used to identify subgroups and longitudinally characterize the gender identity and gender-role behavior of TGD children. These models will define longitudinal patterns of gender identity stability and characterize the relationship between TGD classes and mental and behavioral health outcomes, including the moderating role of social gender transition (when present), on these associations.

**Results:**

Baseline data collection (N=248) is complete, and the identification of TGD subgroups based on gender identity and expression using latent class analysis is anticipated in 2024. The completion of all 4 waves of data collection is anticipated in July 2024, coinciding with the start of a no-cost study extension period. We anticipate longitudinal analyses to be completed by winter 2024.

**Conclusions:**

Through a longitudinal observational design, this research involving prepubertal TGD children and their caregivers aims to provide empirical knowledge on gender development in a US sample of TGD children, their mental health symptomology and functioning over time, and how family initiated social gender transition may predict or alleviate mental health symptoms or diagnoses. The research findings have promise for clinicians and families aiming to ensure the best developmental outcome for these children as they develop into adolescents.

**International Registered Report Identifier (IRRID):**

DERR1-10.2196/55558

## Introduction

### A Call for Lifespan Research on Gender-Diverse Children

Increasing numbers of prepubertal transgender, nonbinary, and gender-diverse (TGD) children, asserting and expressing a gender identity outside of culturally defined norms related to their sex registered at birth [1], are presenting to pediatric gender clinics across the United States and abroad [2-4]. Prepubertal TGD children continue to remain a poorly understood and understudied population in the United States. This trend remains despite a 2011 National Academy of Medicine (formerly the Institute of Medicine) [5] report, “The Health of Lesbian, Gay, Bisexual, and Transgender People,” which explicitly called for longitudinal cohort studies that incorporate a life-course perspective to examine experiences of gender-diverse individuals across developmental stages.

### Gender Dysphoria Prevalence and Course Among TGD Youths

A subset of TGD children experience gender dysphoria (GD), distress that arises from the incongruence between gender identity and sex registered at birth [6]. In 2013, the fifth edition of the *Diagnostic and Statistical Manual of Mental Disorders* (Fifth Edition; *DSM-5*) replaced the now-obsolete diagnosis of gender identity disorder (GID) with GD [6]. In contrast to GID, GD presented greater diagnostic specificity by shifting toward gender identity incongruence and away from simply “cross-sex” behavior by requiring both (1) incongruence between gender identity and sex registered at birth and (2) the presence of distress associated with gender incongruence. Implied by these more stringent GD diagnostic criteria is that some proportion of children assessed and studied in the era of GID may have been falsely diagnosed. Yet, in the *DSM-5*, the theorized etiology, epidemiology, and course of GD were informed by older studies of clinic-referred samples of Dutch and Canadian children who met GID diagnostic criteria [7,8].

Nonetheless, in the absence of research based on the more recent GD criteria, conclusions from research based on GID have been influential in guiding clinical decisions. One oft-cited statistic is that most prepubertal children with GID no longer met criteria for the condition by adolescence or adulthood, and instead developed to be cisgender and sexual-minoritized individuals (eg, gay and lesbian); likely an artifact of the less stringent GID criteria [8-12]. Another finding suggested that only children who satisfied most or all GID diagnostic criteria were more likely to “persist” in endorsing these symptoms in mid to late adolescence thereby meeting treatment eligibility for gender-affirming hormone therapy [9]. TGD children are not eligible for gender-affirming medical interventions (ie, pubertal suppression and gender-affirming hormone therapy) prior to Tanner stage 2 when the first physical signs of puberty occur according to the objective classification system by which physicians track pubertal development [13-15]. The validity of findings from those studies has since been questioned due to notable sampling limitations and methodology for measuring and evaluating gender status in prepubertal children [16]. Concerns have been raised that the absence of more recent research on GD has yielded proliferate misinformation that informs legislative efforts to challenge gender-affirming policies within schools or health care [17].

This study is designed to longitudinally examine gender development and GD (when present) among TGD. Such findings would significantly inform clinical perspectives that may, in turn, inform treatment eligibility standards of gender-affirming medical care for youths with GD who have entered puberty (eg, pubertal suppression and gender-affirming hormone therapy).

### To Affirm or Not to Affirm: Discordant Clinical Approaches to Prepubertal GD

The perspective of a gender health professional as to (1) the extent to which gender identity among prepubertal TGD children is stable over time and (2) the extent to which GD in prepubescence can be reliably predictive of GD into Tanner 2 and beyond may inform the extent to which they support a caregiver facilitating a prepubertal social gender transition in a pediatric patient. A social gender transition is an approach led by caregivers in an effort to address GD in transgender and nonbinary children through various reversible social interventions (eg, by changing the first name, gender pronouns, and manner of dress and grooming) [14]. Emergent empirical findings suggest potential mental health benefits of social gender transition in prepubertal transgender and nonbinary children [18,19]. Informed by this research, as well as expert consensus, the current standards of the World Professional Association for Transgender Health support social transition for prepubertal TGD children on an individualized basis, with the understanding that gender identity can evolve and change over time [13]. However, no known research has observed mental health effects of social gender transition, when present, at more than 3 timepoints across childhood.

### Social Gender Transition and Its Mental Health Correlates

In the absence of longitudinal outcomes on psychosocial health, prepubertal social gender transition remains controversial. This controversy may be further sustained by findings from the previously mentioned research that imprecisely examined children with GID and spuriously suggested that, in most cases, these children did not continue to experience GID upon reaching puberty [20,21]. Those unaware of these studies’ limitations may believe without question that most prepubertal children do not continue to assert a transgender or nonbinary identity beyond puberty and that a prepubertal social gender transition is premature and a potential source of emotional harm when a child wishes to transition back to a gender consistent with their sex registered a birth. However, 2 cross-sectional studies of transgender and nonbinary children aged 3-12 years [19] and 6-14 years [18] suggest that social gender transition may be a psychosocially protective factor for transgender and nonbinary children. In both studies, nonclinical samples of transgender and nonbinary children who underwent family initiated social gender transition exhibited age-normative depressive and anxiety symptoms with anxiety rates slightly higher than controls (ie, cisgender siblings and age-matched controls) but still well below a clinical level. Beyond these cross-sectional studies that used cisgender children as comparison groups, there is a need to longitudinally examine mental and behavioral health outcomes in a “within-group” fashion. For example, it is important to examine the mental health outcomes of prepubertal transgender and nonbinary youths who are socially transitioned compared to those who are not.

### Mental Health Correlates Among TGD Children

Beyond the existing studies is the need to longitudinally examine mental and behavioral health outcomes (both positive and negative) in prepubertal TGD children in the United States including the extent to which family rejection or support factors may moderate these outcomes. The existing body of scientific evidence documenting the health and well-being of prepubertal TGD children is sparse, but studies to date suggest transgender and nonbinary children may experience and be at risk for developing mental health problems. One Canadian study found that 47% to 89% of transgender and nonbinary children experienced problem behaviors in the clinical range, per caregiver report [21]. Another study found that 52% of prepubertal TGD children with GID met diagnostic criteria for at least one additional psychiatric condition, more often internalizing (anxiety, depression) than externalizing (attention-deficit/hyperactivity disorder, oppositional defiant disorder) in nature [22]. Several factors have been found to contribute to these co-occurring conditions including lack of family acceptance and support, family risk factors for mental health problems, and peer or community social ostracism. Consistent with this formulation, numerous studies of transgender and nonbinary adolescents have suggested that distress related to GD may contribute to disproportionate psychosocial adversity including depressive symptoms, low life satisfaction, self-harm, isolation, homelessness, incarceration, posttraumatic stress, and suicidal ideation and attempts [23-27]. Without appropriate support, as they age into young adulthood, transgender and nonbinary youths are likely to face economic and societal marginalization, incarceration, and physical abuse leaving them at significantly higher risk for substance use, violence, acquisition of HIV, other sexually transmitted infections, and homelessness [23,25,28,29]. Attention must be placed on research that can inform clinical practice early in the developmental process with all prepubertal TGD children, possibly serving as preventive interventions in the face of these later risk factors.

### Gender Journeys: Gender Identity Development in Prepubertal TGD Children

A dearth of empirical evidence exists on the extent to which prepubertal TGD children understand their gender. Gender constancy refers to a child’s ability to report their gender identity stably and consistently over time, irrespective of changes in gender-typed activities or appearances [30]. Based on developmental research to date (and samples presumed to consist of non-TGD children), gender constancy is considered to be achieved by age 4-7 years, and it is foundational to the formation of knowledge structures (ie, gender schemas) that inform how children learn gender-role norms and mores [31]. At the time of this study protocol’s development, only one known study assessed gender cognition in prepubertal TGD children, using a cross-sectional design, and found that transgender and nonbinary children were comparable to cisgender controls in gender-cognition ability [32]. This finding suggested that prepubertal transgender and nonbinary children may reach gender development milestones at periods similar to those theorized among cisgender children. Nonetheless, without longitudinal observation of prepubertal TGD children, there is no empirical basis for understanding important milestones of gender development in these children. The features of these milestones include the extent to which patterns of gender identity development, gender expression, and role behavior are stable over time, and what factors predict continuity or change in these patterns as children age.

### Responding to a Call: A Study of Prepubertal GDC

This study’s principal investigators (PIs; ie, MAH, DC, and DE) and coinvestigators (ie, RG, JO-K, and SMR) comprise a multidisciplinary team of clinician-scientists affiliated with the 4 enrolling sites. These sites are US gender centers considered national leaders delivering similar models of multidisciplinary pediatric care and include the University of Southern California (USC) and the Center for Transyouth Health and Development at Children’s Hospital Los Angeles (CHLA; MAH, formerly and JO-K), Northwestern University (NU) and the Gender & Sex Development Program at Ann & Robert H. Lurie Children’s Hospital of Chicago (LCH; DC and RG), University of California San Francisco (UCSF) and its Child and Adolescent Gender Center at Benioff Children’s Hospital (DE and SMR), and University of California Los Angeles (UCLA) and its Gender Health Program at UCLA Health (MAH, presently). An additional site—the Gender Multispecialty Service at Harvard Medical School’s Boston Children’s Hospital (HMS/BCH; ACT and DC)—withdrew in year 2, prior to initiation of enrollment, due to the absence of a qualified behavioral clinician-scientist PI to replace the original site PI (ACT), who had relocated to a liberal arts university with no gender clinic affiliate. All authors are veteran investigators in Trans Youth Care United States (TYCUS), an ongoing, longitudinal, observational study of psychosocial and anthropometric outcomes of gender-affirming medical care among TGD adolescents (R01HD082554-06A1). With this adolescent cohort research already underway, our study team was well-suited to recruit a cohort of prepubertal TGD children in further response to the 2011 National Academy of Medicine Report calling for rigorous lifespan research aimed at understanding the health implications of approaches to gender-affirming care.

### Gender Journey Project’s Overarching Goal and Specific Aims

The goal of this study, Gender Journey Project, is to provide evidence to inform clinical care for prepubertal TGD children. We are pursuing 3 scientific aims over an 18-month observational period, collecting behavioral data from prepubertal TGD children and a primary caregiver at 6-month intervals to (aim 1) describe characteristics of gender identity and expression in a cross-section of prepubertal TGD children to inform clinical classification and care, (aim 2) describe and characterize longitudinal patterns of gender identity development and gender expression to inform the timing of clinical intervention, and (aim 3) longitudinally characterize the relationship between identity-expression-dysphoria profiles and mental and behavioral health outcomes—both negative (eg, GD and externalizing behaviors) and positive (strengthened resilience and social confidence)—and the moderating role of social gender transition (when present) on these relationships.

## Methods

### Study Design

This ongoing study uses a longitudinal, observational multisite design to better understand prepubescent trajectories of gender diversity (identity and behavior), GD, mental health, and well-being among a cohort of TGD children. Participants include a TGD child and 1 caregiver who were studied prospectively over an 18-month period.

### Community Engagement

At several junctures throughout the study period, our team used community-engagement methods in the form of Key Informant Advisory Boards, a participant feedback survey, and a feedback item in the finalized child and parent surveys to help tailor methods of the study protocol to our target population while also seeking to limit participant burden. The 3 separate Key Informant Advisory Boards were each comprised of a distinct group: TGD youths (slightly older than our target population but who may have been eligible for participation if not for their age), caregivers of TGD youths, and licensed mental health clinicians with expertise in pediatric gender health. Board members provided input on the study visit structure and measure selection; youth members named the study. In the first 8 weeks of baseline data collection, immediately after completing their baseline study visit, 12 caregivers and 7 child participants agreed to complete a 15-minute participant feedback survey for which they were each paid US $10 via a reloadable cash card (eg, ClinCard) that, although issued to the caregiver, is shared by the child and caregiver. Caregivers were eligible to participate without their child and vice versa. The survey objective was to elicit participant feedback on several aspects of study participation (eg, screening and informed consent or assent procedures, usability of the computerized and survey-delivery format, basic understanding of the study content, and the overall sequencing of the study visit). Finally, in the subsequent survey versions used at all study periods, we included an open-ended item to capture additional participant feedback.

### Study Population and Recruitment

A total of 248 prepubertal TGD children and their caregivers are enrolled in the study. Participants were recruited nationally through a number of pathways, including seeking gender-affirmative mental health care for GD at any of the 4 affiliated sites, referred from local community providers, or self-referred. Initially, we anchored enrollment to the 4 sites (and community referrals local to each) before eventually widening recruitment nationally to help meet enrollment goals in the final year.

To be considered eligible for enrollment, children must have met criteria that include: residing in the United States, a caregiver-reported history of gender-nonconformity for at least the past 6 months or a GD diagnosis per *DSM-5* criteria, Tanner stage 1 pubertal development (ie, lack of pubertal development) per structured prescreening by caregiver or child, aged 6-13 years, the ability to understand English, access to a computer with webcam, microphone and internet capabilities, willingness to provide informed assent, and a caregiver willing to provide caregiver permission for the child to participate. The exclusion criteria include prior use of gonadotropin-releasing hormone agonist to suppress puberty, the presence of serious psychiatric symptoms that would impair abilities to assent or complete baseline, or being visibly distraught. Adults considered eligible must: be a primary caregiver of the child participant, be at least age 18 years, have the ability to read and understand English, have access to a device with internet capabilities, and have the ability to provide informed consent on their and the child participant’s behalf. Caregiver exclusion criteria include the presence of serious psychiatric symptoms, being visibly distraught, and being under the influence of alcohol or substance use.

### Ethical Considerations

The institutional review board (IRB) of the record is UCLA (10-00029) through a single-IRB agreement established to include USC/CHLA, NU/LCH, and UCSF. Prior to the primary author’s (ie, Contact PI) relocation to UCLA from USC/CHLA in the third quarter of the project period’s third year, IRB approval was granted to NU/LCH and UCSF under a reliance agreement established through the USC/CHLA IRB (19-00108). Study staff members conducted informed consent and assent procedures with each child-caregiver dyad via video-conferencing software. These procedures consisted of reviewing respective forms to, first, obtain child assent, and then, caregiver consent. The caregiver was present for both assent and consent procedures, while children were given the option to stay or be dismissed once caregiver consent procedures began. Children and caregivers signed forms electronically using the electronic signature tool. All enrollment and survey data collected is electronically stored, under policies compliant with the Health Information Portability and Accountability Act and the Health Information Technology for Economic and Clinical Health Act. Child and caregiver participants are each provided US $25 after completion of their respective study visits. These reimbursements are disbursed to a reloadable cash card immediately following the completion of the study visit.

### Data Collection and Measures

#### Overview

To accommodate public health social-distancing requirements, study visits are all conducted remotely. Caregiver report measures are collected remotely through a computer-assisted self-completed survey. Child report measures are collected remotely through a computer-assisted survey conducted by a research associate over a video-conferencing platform.

#### Domains Common in Child and Parent Surveys

Child and caregiver surveys are completed at baseline, 6, 12, and 18 months. Both surveys include domains assessing the child participant’s GD-specific experiences (including feelings about primary sex characteristics and other aspects of anatomy), mental health, and social relationships. Caregiver participants completed the survey in 40-65 minutes. Child participants completed the survey in 50-90 minutes, due to the increased time of the child survey’s verbal administration, as well as multiple 3- to 5-minute breaks issued to the child throughout. Caregiver and child participants complete their respective surveys privately and typically not during the same visit because the caregiver participant is advised to be available to research associates throughout the child’s remote study visit if needed. Caregiver participants are given up to 72 hours after the child’s study visit is completed to complete the caregiver survey.

#### Domains Specific to Child Survey

Unique to the child survey is a domain related to implicit gender cognition. At the advice of community informants, child participants are only asked items pertaining to the child’s suicidality and perceptions of anatomy if, during informed assent, the child and their caregiver elect for the child to receive these items during the survey. In cases when suicidality items are endorsed in either the caregiver or child surveys, a research associate checks in with participants to determine whether professional mental health support is needed and whether the study visit should continue or be postponed. At the end of the visit and at the start of the subsequent study visit 6 months later, an additional check-in occurs regarding child safety.

#### Domains Specific to Caregiver Survey

Unique to the parent survey are additional domains related to caregiver stress, caregiver support related to child gender diversity, medical information about the child, the impact of COVID-19 on the family, and demographics. Caregivers also complete the Brief Problem Monitor for Ages 6-18 [33], an empirically validated measure for rating children’s internalizing and externalizing problems.

### Statistical Analysis

#### Overview

Existing research on TGD youths largely uses variable-centered approaches to data analysis (eg, regression, factor analysis, and structural equation modeling) with a focus on relationships among variables (eg, to predict outcomes) to study how indicators are related to latent constructs (eg, factors) and how constructs relate to independent and dependent variables. More recently, research on TGD youths has capitalized on advances in person-centered methods using latent class analysis (LCA) [27,34-36]. The conceptual impetus for this shift is that individuals may differ in their development and timing as to when they assert or express diverse gender identities. Informed by the recognition that person-centered methods are of high utility for epidemiological and clinical research by allowing the effects of risk factors, outcomes, as well as treatment modalities, to vary across subgroups of youths, latent class models will serve as the primary analytic method in this study.

#### Aim 1: Characteristics of Gender Identity and Gender-Role Behavior Among Prepubertal TGD Children to Inform Clinical Classification and Care

**Figure 1:**
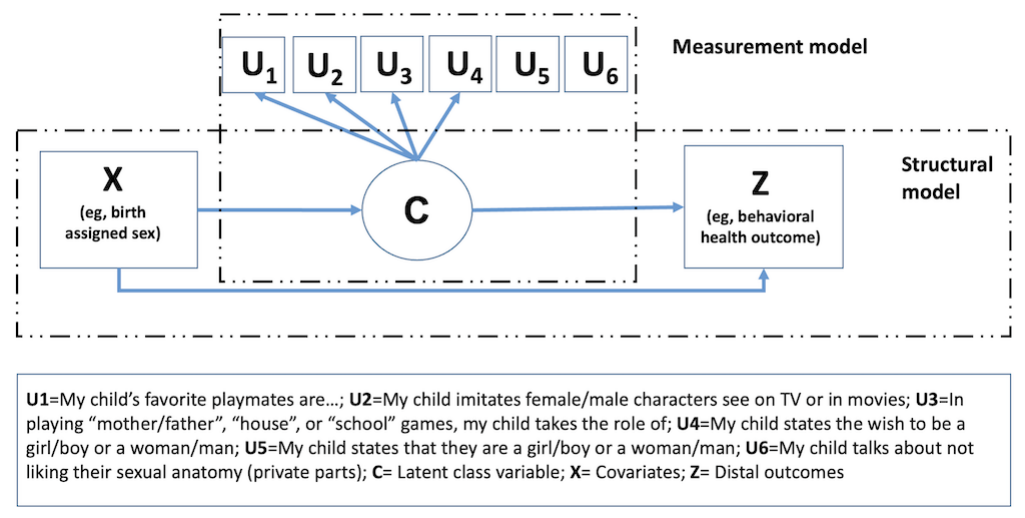
A latent class model with covariates (X) and a distal outcome (Z). C: latent class variable; U: latent class indicators; X: covariates; Z: distal outcomes.

To address this aim, separate latent class models will be estimated for caregiver and child ratings of gender identity and gender expression using indicators from the gender development scale (J Strang et al, unpublished, 2016). An LCA describes how the probability of endorsing a set of observed categorical variables or indicators may vary across groups of individuals where group membership is not observed [37-39]. For example, youths who are transgender and identify as either male or female (ie, binary transgender youths) are hypothesized to endorse different indicators than nonbinary youths. For this aim, we focus on the measurement model only (ie, the relationship between the gender identity and expression indicators and the categorical latent variable). Using a structural equation modeling approach, the circle in Figure 1 refers to a categorical latent variable representing the discrete number of latent groups and the boxes represent observed variables, which can serve as (1) latent class indicators “U” (ie, gender identity indicators), (2) predictors of class membership “X” (eg, sex registered at birth), or (3) distal outcomes “Z” (eg, mental and behavioral health outcomes) associated with each latent class. As mentioned earlier, group membership itself is not an observed variable. To this end, models with an increasing number of groups are estimated and compared based on recommended fit indices [40-42]. In addition to statistical fit indices, model selection will also be informed by the substantive interpretability of the groups, as well as considerations of parsimony with respect to the number of model parameters. A selected latent class model will indicate the “optimal” number of groups, the number of individuals in each group, and group-specific response profiles for parent and child ratings.

#### Aim 2: Characterize Longitudinal Patterns of Gender Identity Development and Gender-Role Behavior to Inform Timing of Clinical Intervention

**Figure 2:**
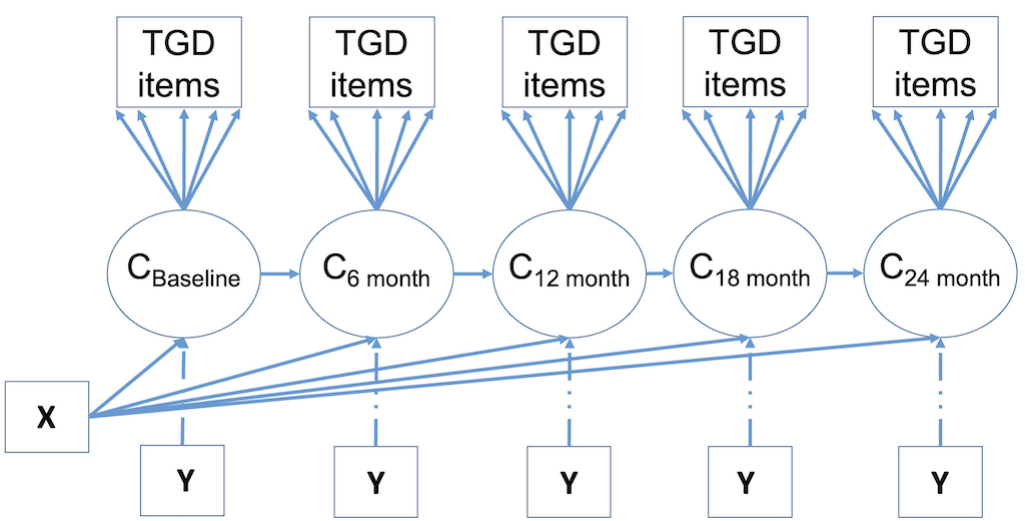
Latent transition model with time-invariant (X) and time-variant (Y) covariates. TGD: transgender, nonbinary, and gender-diverse.

To address this aim, a longitudinal extension of LCA will be implemented, namely latent transition analysis (LTA) [43-45] (Figure 2 [43]). Building upon the timepoint-specific LCA models, models from 2 or more waves are linked to study individual transitions from a gender diversity profile at 1 wave to the same or different profile at the subsequent wave. Generally, transitions can be described in terms of “stayers” (ie, transitioning to the same profile at a later wave) and “movers” (ie, transitioning to a different profile at a later wave). We hypothesize two types of movers, that is, (1) those who transition to a profile that was observed at a prior wave, and (2) individuals who transition into a profile that is newly evolved at a later wave. LTA models can be estimated after 2 rounds of data collection. When more than 2 waves of data are available, the lag-1 model can be extended to assess other lag models. For example, it is possible to explore whether profiles at baseline directly influence profiles at 12 months (ie, 2 timepoints later). In addition, it is important to assess whether transition probabilities are stationary (constant across waves) or whether there is evidence for more likely transitions occurring at certain time points, for example, due to pubertal onset after baseline. Another reason certain transitions may be more likely is the possibility that families may make a treatment decision to suppress or “pause” the child’s pubertal development during the course of this study, or potentially support a social gender transition. Such influences can be further explored by adding covariates to the model, as discussed in more detail below.

#### Aim 3: Longitudinally Characterize the Relationship Between Identity-Expression-Dysphoria Profiles, Mental and Behavioral Health Outcomes, and the Moderating Role of Social Gender Transition (When Present) on These Relationships

To address this aim, selected mental and behavioral health variables are included as outcomes in the wave-specific LCA models (Figure 1). As discussed earlier, the inclusion of outcomes is part of a comprehensive substantive model assessment strategy. If distinct TGD child profiles do not differ with respect to outcomes, their theoretical fit must be questioned. To ensure the correct timing, LCA profiles are related to outcomes measured at *t*+1. This model essentially resembles a linear (for continuous outcomes) or logistic (for categorical outcomes) regression model with a categorical latent predictor variable (ie, TGD child profiles). In addition, the effect of outcome differences by latent class profile can be assessed in conjunction with outcome differences by covariates (Figure 1). This can help to differentiate the contribution covariates and profiles make in explaining differences in outcomes. In other words, we can assess whether covariates at *t*–1 explain or overwrite any differences in outcomes associated with profile membership. For this scenario, a latent class profile at *t*+1 is simultaneously regressed on the baseline profile and a baseline predictor variable. Both effect paths are parameterized by a multinomial logistic regression. It is furthermore possible to assess the extent to which the influence of covariates is moderated as a function of profile membership. For example, we can assess the effect of family initiated social gender transition on mental health outcomes for each latent class profile of TGD children.

#### Power

To arrive at credible estimate inputs for the power calculations, data from a small sample (n=34) were analyzed. These data were drawn from measures administered as part of the standard of care for all patients aged 12 years and younger who presented to the Lurie Children’s/NU multidisciplinary Gender Development Clinic, a subspecialty clinic established in July 2013. Latent class analyses identified 3 distinct groups. Of the 34 cases in the sample, 21% (n=7) fit with what we termed a “gender fluid” profile (ie, moderate levels of gender-diverse behavior and low to moderate levels of gender identity incongruence), and 27% (n=9) fit with what we termed a “gender-nonconformity” profile (ie, high levels of gender diverse behavior and low degree of gender identity incongruence). The largest group (n=18, 53%) consisted of youths with both high levels of gender-nonconforming behavior and high levels of gender identity incongruence (ie, transgender youths).

A minimum sample size of 100 is needed to detect a medium effect of a continuous covariate on both gender identity profiles. With a sample size of 248, a small effect on membership in the gender nonconformity group can be detected. A minimum sample size of 200 was needed to detect a medium effect of a binary covariate on membership in the gender nonconformity group. To detect a medium effect for the gender-fluid group, a minimum sample size of 300 is required. In summary, with a targeted sample size of 248, the proposed study is sufficiently powered to detect, at minimum, a medium covariate effect for membership in both profiles. Since full information maximum likelihood estimation will be used, all youths who contribute information for at least 1 wave will be included in the analysis and thus loss to follow-up does not alter the conclusions from this power analysis [46].

## Results

### Baseline Sample

When originally proposed, sites in each of the 4 cities were to recruit 80 cases to contribute to the overall sample (N=320). Upon withdrawal of the Boston site, sites in the remaining 3 cities were responsible for recruiting a one-third greater sample than their original target. Study recruitment closed in November 2022, and the last baseline survey was completed in January 2023. Our baseline sample includes 248 child participants (78% of our original target of n=320). The average age of the child baseline sample is 8.42 (SD 1.75; range 6-13) years. Of the child participants, 69% (n=171) are registered male at birth and 31% (n=77) are registered female at birth. Preliminary child race or ethnicity data were collected from caregivers and indicate that 52% (n=128) of the sample is non-Hispanic or non-Latine White; 14% (n=34) of the sample is Hispanic or Latine; 17% (n=43) of the sample is bi- or multi-racial; and, non-Hispanic or non-Latine Black (n=1) and Asian (n=1) participants each comprised less than 1% of the sample. Race and ethnicity data for 34 participants (14%) were not reported by caregivers. Of the caregiver sample, 89% (n=214) of the sample is registered female at birth and 12% (n=30) of the sample is registered male at birth, with about 2% (n=4) not reporting registered sex at birth. All intervals of study visit follow-up are now underway.

### Extenuating Challenges to Study Enrollment

We consider it a great success to enroll 248 child and parent participants given several challenges that coincided with enrollment efforts. First, were the many emergent and muddying challenges associated with the COVID-19 pandemic, the onset of which coincided with the original launch of study enrollment. Originally, the study procedures were to occur entirely in person at research facilities located at each study site. A child participant and the caregiver participant were to complete their respective surveys privately during the same visit. Significant delays were borne out of the subsequent quarantine, the overall “new normal” of socially distanced interaction, and fit-and-start patterns that emerged alongside new viral variants. The most notable challenges included institutional months-long halting of human participant research, as well as staffing shortages that affected the productivity of IRB and research compliance offices; a 6-month delay in starting data collection to overhaul study procedures from entirely in-person to entirely remote; and delays in visits due to illness of a child participant, a caregiver participant, or a study staff member. Second, was the withdrawal of the Boston site (ie, HMS/BCH) at the end of year 2. At that point, HMS/BCH had not started enrollment due to delays in obtaining IRB reliance with USC/CHLA. Consequently, its withdrawal significantly increased recruitment demand at the 3 remaining sites. Third, was a 6-month pause in recruitment during the relocation of the Contact PI and coordinating center from 1 Los Angeles academic medical center with an affiliated gender program (ie, USC/CHLA) to another (UCLA).

## Discussion

### Principal Product

This paper describes the protocol of a research study that seeks to longitudinally observe a national cohort of prepubertal TGD children (and a caregiver) with the aim of expanding empirical knowledge pertaining to gender development and cognition in prepubertal TGD children, their mental health and functioning over time, and how family initiated social gender transition may predict or alleviate mental health symptoms. This study will use state-of-the-art measures and use statistical advances in person-centered analytical approaches (ie, LCA and LTA) to address inconsistencies in prior studies, which have focused primarily on variable-centered approaches. This study expands on the current collaboration of TYCUS study investigators to include a younger and understudied cohort of prepubertal TGD children. The average age of our 248 baseline child participants is 8 (SD 1.75) years, the sample is predominately White (non-Hispanic or Latine), and over two-thirds registered male at birth.

An analysis of sample characteristics is beyond the scope of this protocol paper, but ostensibly, the sample resembles clinic-derived samples of TGD children which were largely White and with more registered males at birth referred for care at younger ages and more often than registered females at birth, with ratios ranging from 6:1 (Canada), 4:1 (the United Kingdom), 3:1 (the Netherlands), and 2:1 (NU/LCH) [2,47]. Although our sample is not clinic-derived, its composition may be similar to that of previous studies given its affiliation with prominent 4 academic gender health centers.

### Limitations

The protocol described here is not without limitations. It is certainly a limitation that our sample is somewhat racially homogeneous, which may present challenges to how we evaluate our hypotheses within and between racial subgroups. In addition, in collecting data from and about only 1 caregiver, we are limited in our ability to examine the effects of attitudes and influences of additional caregivers (when present) on our outcomes of interest. Last, given the observational design of our study, its findings will have limited generalizability.

### Conclusions

Limitations aside, the current researchers anticipate expanding the investigation into a program of research that examines the experiences and needs of TGD youths from early childhood through early adulthood. This study sets up an ideal framework to continue collecting longitudinal data from the cohort recruited for this initial work, and further elucidate the developmental complexities of this population to complement findings from the TYCUS study.

## Abbreviations

BCH: Boston Children’s Hospital
CHLA: Children’s Hospital Los Angeles
DSM-5: Diagnostic and Statistical Manual of Mental Health Disorders (Fifth Edition)
GD: gender dysphoria
GID: gender identity disorder
HMS: Harvard Medical School
IRB: institutional review board
LCA: latent class analysis
LCH: Lurie Children’s Hospital
LTA: latent transition analysis
NU: Northwestern University
PI: principal investigator
TGD: transgender, nonbinary, and gender-diverse
TYCUS: Trans Youth Care United States
UCLA: University of California Los Angeles
UCSF: University of California San Francisco
USC: University of Southern California

## Data Availability

The data sets generated by and to be analyzed during this study are not yet publicly available due to ongoing data cleaning or due to not having yet been completed but are available from the corresponding author upon reasonable request.
